# Awareness and willingness to utilize HIV pre-exposure prophylaxis and associated factors among men who have sex with men in Maanshan, China

**DOI:** 10.1371/journal.pone.0324259

**Published:** 2025-05-22

**Authors:** Quan Fang, Gan Tang, Ziwei Wang, Qian Guo, Qisheng Guo, Yinguang Fan, Qirong Qin

**Affiliations:** 1 Lishui Center for Disease Control and Prevention, Lishui, Zhejiang Province, PR China; 2 Department of Epidemiology and Biostatistics, School of Public Health, Anhui Medical University, Hefei, Anhui, China; 3 Ma’anshan Center for Disease Control and Prevention, Ma’anshan, Anhui Province, PR China; University of Technology Sydney, AUSTRALIA

## Abstract

**Objective:**

Men who have sex with men (MSM) are disproportionately affected by HIV and represent the primary target population for pre-exposure prophylaxis (PrEP) use. However, PrEP adoption in China remains limited, partly due to its late regulatory approval. This study aims to investigate the awareness and willingness of PrEP use and associated factors among MSM in Maanshan city, so as to promote the popularization of PrEP.

**Methods:**

This cross-sectional study was conducted in Maanshan City, China, between June 2016 and December 2019. Participants completed the questionnaire through respondent-driven sampling (RDS). The questionnaire information was organized and analyzed using SPSS 23.0 software. Both univariate and multivariate logistic regression models were employed to investigate the determinants of PrEP willingness and awareness among HIV-negative MSM.

**Results:**

A total of 879 participants were enrolled, with 837 providing analyzable data. The majority (62.25%) were aged <30 years old, with 97.49% self-identified as homosexual. Among participants, 50.18% reported regular male sexual partners. Regarding sexual behaviors, 71.80% of MSM engaged in casual sex and 36.56% unprotected anal sex (UAI) within the last six months. HIV awareness was reported by 92.83% of respondents, while PrEP awareness was substantially lower (22.70%). Willingness to use PrEP was high (89.49%), with 16.49% preferring to take PrEP daily and 84.59% preferring to take PrEP on demand. Multivariate logistic regression revealed that vocational school (vs. high school or below), recent casual sex engagement, PrEP awareness, and recent UAI history were significant predictors of PrEP willingness. Higher education (university or above vs. high school or below), versatile sexual role (vs. op/insertive ones), recent casual sex and prior HIV testing were positively associated with PrEP awareness.

**Conclusions:**

Maanshan’s MSM population demonstrated high PrEP acceptance but limited awareness. The findings suggest that expanding access to HIV testing could enhance PrEP awareness. Simultaneously, PrEP dissemination combined with targeted HIV prevention may effectively reduce HIV transmission in the MSM population.

## Introduction

Men who have sex with men (MSM) — a term encompassing gay, bisexual, and other men who engage in male-male sexual activity regardless of self-identified sexual orientation — are at elevated risk of HIV infection due to behavioral factors such as multiple sexual partnerships and unprotected sex [1]. In China, HIV transmission has shifted from the general population to high-risk groups [2], with the proportion of male-to-male sexual transmission increasing from 2.5% in 2006 to 25.6% in 2022 [3]. Thus, preventing HIV among high-risk populations, particularly MSM, remains a critical public health challenge. Pre-exposure prophylaxis (PrEP), a biomedical intervention involving daily or on-demand antiretroviral use by HIV-negative individuals, has demonstrated high efficacy in reducing sexual HIV transmission [4]. When adhered to consistently, PrEP with tenofovir disoproxil fumarate (TDF) and emtricitabine (FTC) reduces HIV acquisition risk by over 90% among MSM [5,6]. Studies have shown that MSM in many countries had limited awareness of PrEP but were highly willing to use it [7–10]. However, in China, PrEP uptake has been constrained by cost, adherence challenges, and potential risks [11]. Although some studies indicate that PrEP acceptance rate among MSM in China ranges from 30.28% to 72.4% [12]. However, due to the late introduction of PrEP in China, the number of relevant studies remains limited. Variations in regions, high-risk populations and medication preferences indicate that the large-scale PrEP promotion in China is both feasible and promising [13].

Maanshan, a city in eastern Anhui Province, has strong geographical, economic, and cultural ties with the cities of the Yangtze River Delta region. Frequent Inter-regional mobility among MSM facilitates easier access to sexual partners and increases exposure to diverse male populations with varying HIV subtypes and HIV endemic patterns [14]. This dynamic raises the risk of HIV and other sexually transmitted infections. The HIV infection rate among MSM in Maanshan is 12.6%, higher than in neighboring Nanjing (8.3%) [15,16]. The population mobility may affect the worldwide spread of HIV in Nanjing. However, it was similar to Chengdu (15.5%), a key HIV reporting hotspot in China [17]. Additionally, over half of MSM in Chengdu demonstrate high PrEP awareness [15–19]. A Danish study further demonstrates PrEP use — particularly during international travel — effectively supports HIV prevention both domestically and abroad [20]. Given Maanshan City’s well-connected transportation network, it is critical to assess the local impact on PrEP knowledge and strengthen public awareness of HIV exposure prevention. Since the first case of AIDS was reported in Maanshan in 1999, the overall prevalence has steadily increased [21].

PrEP was not covered in China’s national health insurance until 2017 and only received approval from the China National Medical Products Administration for HIV risk reduction in 2020 [22]. Moreover, no data currently exist on PrEP usage prevalence among MSM in Maanshan City. This study therefore aims to evaluate awareness, willingness to use PrEP and associated factors among MSM in Maanshan during this period. The findings will provide crucial baseline data to guide targeted interventions and public health strategies for improving PrEP uptake among in this population.

## Materials and methods

### Study design and population

**Fig 1:**
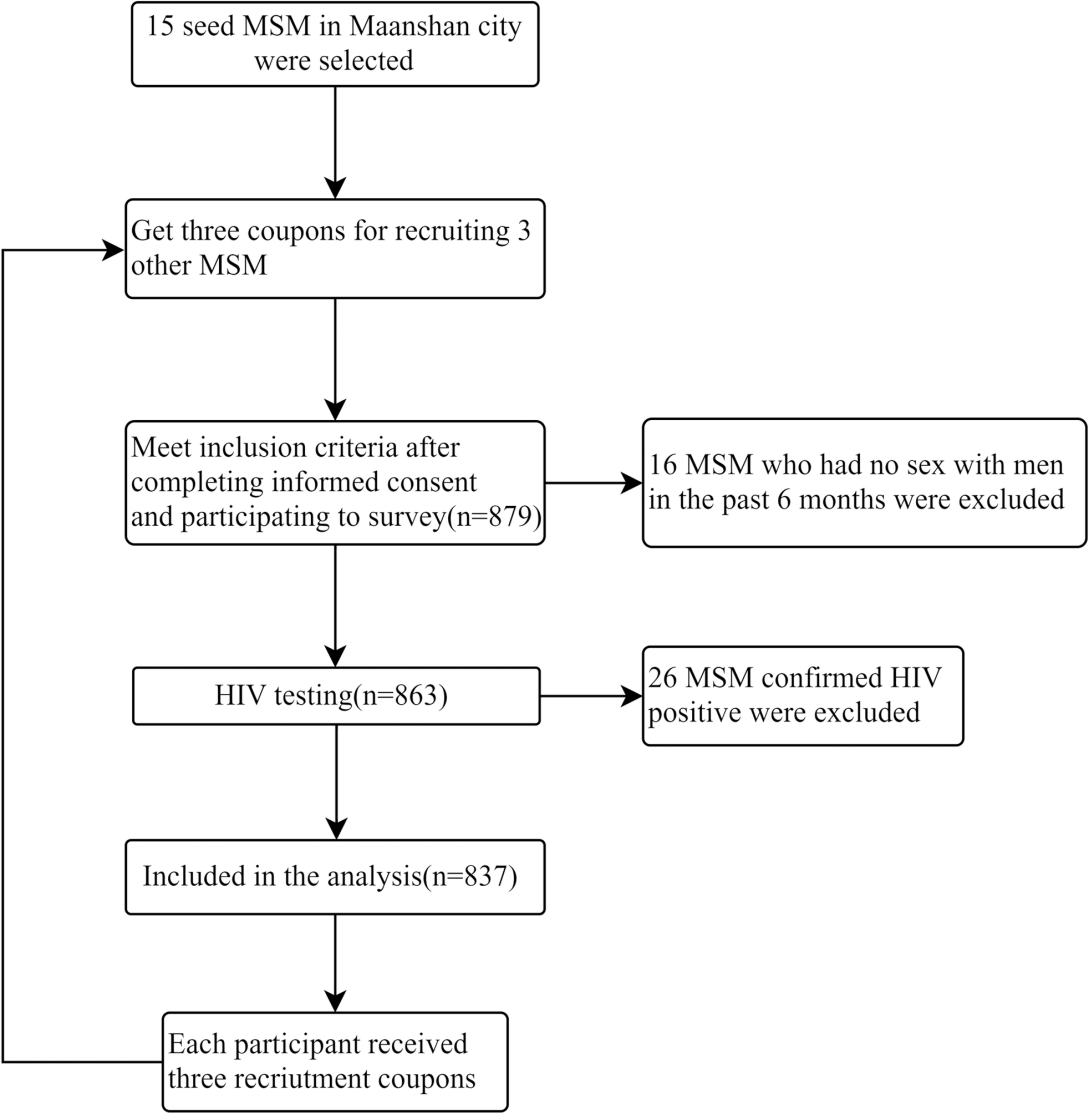
Flow chart of recruitment.

In this study, MSM participants were recruited with assistance of staff from the Maanshan CDC. This cross-sectional study was conducted in Maanshan City, China between 1 June 2016 and 31 December 2019. Participants completed the questionnaire via respondent-driven sampling (RDS), a chain-referral method. The initial 15 participants (“seeds”) were selected by MSM-friendly organizations and given three coupons each to recruit three additional MSM. For each successful referral enrolled in the study, participants received an incentive of 20 CNY (approximately 3.5 USD). Recruitment continued for up to five waves (e.g., participant 1 recruits Participant 2, who recruits Participant 3, etc.), resulting in a final independent sample (Fig 1).

The inclusion criteria for the participants were: 1) male; 2) aged 18 years or older; 3) current resident of Maanshan City at the time of the survey; 4) had sex with a man in the past six months; and 5) received HIV test results and follow-up advice. Participants were excluded if they were: 1) diagnosed with a mental illness; 2) Previously diagnosed with HIV infection; 3) unable or unwilling to provide written informed consent. All recruiters had rigorous training and regularly reviewed the questionnaire for accuracy. The study was approved by the Ethics Review Committee of Anhui Medical University (NO.20131193).

### Outcomes

The study assessed two primary outcomes: PrEP awareness and willingness to use PrEP. PrEP awareness was assessed by four questions:1) “Should PrEP be used when regularly having intercourse with an HIV-positive partner?”, 2) “Is PrEP necessary before having sex with an HIV-negative partner?”, 3) “Should PrEP be used when having sex with a casual partner of unknown HIV status?” and 4) “Will you use a condom after taking PrEP?”. Participants were considered aware of PrEP only if they answered “yes” to all four questions. Willingness to use PrEP was measured using the question: “Will you use PrEP if needed?”. Responses were recorded on a 3-point Likert scale:1 = “definitely not”, 2 = “probably”, and 3 = “definitely”. Participants were also questioned about their preferred form of PrEP and, if unwilling to use it, their reasons for declining.

### Variables

The variables in the questionnaire were determined by referring to previous studies and considering the influencing PrEP promotion in Maanshan city [23,24]. All variables were categorized into demographics, sexual behaviors and sexual health-related care services. Demographic characteristics include age, ethnicity (Han ethnicity or minority); education level (high school or below, vocational school, university or above), marital status (never married, married, divorced/widowed), monthly personal income, history of injection drugs and sexual orientation (gay, heterosexual/bisexual/unsure). Sexual behaviors include the sexual role (insertive, receptive, versatile), history of regular male sexual partners, age at first anal sex with a man, number of anal sex acts in the last month, history of casual sex in the previous six months, and history of unprotected anal sex (UAI) in the last six months. Sexual health-related care services include HIV awareness (measured using UNGASS standards, where knowing 6 of 8 questions was considered adequate), PrEP awareness and willingness to use, preferred PrEP delivery method, reasons for not using PrEP, history of HIV testing and past STD infections within the past year.

### Data analysis

Duplicate and incomplete questionnaires were removed and the completed data were entered into a database created using EpiData 3.1 software. Quantitative data were described using medians and interquartile ranges (IQR). Categorical data were described using frequencies and percentages. To examine factors associated with willingness to use of PrEP and PrEP awareness among MSM, univariate and multivariate logistic regression models were used. All variables with p < 0.1 in the univariate analysis were included in the multivariate models. A two-tailed p < 0.05 was considered statistically significant. All analyses were performed using SPSS 23.0 software.

## Results

### Demographic and sexual behavior characteristics

The study initially included 879 participants, of whom 16 were excluded for not having had sex with a man in the previous six months and 26 were disqualified due to HIV-positive status, resulting in 837 eligible participants for analysis. Among these, 5.4% reported having taken PrEP for HIV prevention. Demographic characteristics (Table 1) showed that 62.25% participants were under 30 years old and 61.65% had never married. The vast majority identified as homosexual (97.49%) and reported no history of drug use (99.40%).

**Table 1:** Demographic characteristics and sexual behavior among HIV-negative MSM in Maanshan City, China (N = 837).

Variable	Subgroup	Frequency/Median	Percentage (%)
Total		837	100.00
Age, years	18-25	284	33.93
	26-30	237	28.32
	>30	316	37.75
Ethnicity	Han ethnicity	831	99.28
	Minority	6	0.72
Education	High school or below	393	46.95
	Vocational school	250	29.87
	University or above	194	23.18
Marital status	Never married	516	61.65
	Married	262	31.30
	Divorced/Widowed	59	7.05
Monthly personal income, RMB	<1000	113	13.50
	1000-2999	298	35.60
	≥3000	426	50.90
Have ever taken drugs	No	832	99.40
	Yes	5	0.60
Sexual orientation	Gay	816	97.49
	Heterosexual/Bisexual/Unsure	21	2.51
Sexual role	Top/Insertive	266	31.78
	Bottom/Receptive	148	17.68
	Both of it	423	50.54
Whether have a regular male sexual partner currently	No	417	49.82
	Yes	420	50.18
Age of first anal sex with men^a^		21	(19,23)
Number of anal sex acts in the previous month^a^		2	(1,3)
Casual sex in the last 6 months	No	236	28.20
	Yes	601	71.80
UAI in the last 6 months	No	531	63.44
	Yes	306	36.56

^a^: discribe using medians and IQR.

Regarding sexual roles, 50.54% identified as versatile, 31.78% as top/insertive, and 17.68% as bottom/receptive. Half of participants (50.18%) reported having a regular male sexual partner. The median age at first anal sex with men was 21 years (IQR: 19, 23), with a median frequency of 2 anal sex acts (IQR: 1, 3) in the previous month. Within the last six months, 71.80% reported casual sexual behavior and 36.56% reported unprotected anal intercourse.

### Sexual health care

The study found HIV awareness among respondents was high (92.83%), while PrEP awareness remained considerably lower (22.70%). Nearly 90% of MSM (89.49%) expressed willingness to use PrEP, with 16.49% preferring daily use and 84.59% favoring on-demand regimens. Key barriers to PrEP adoption included: concerns about the medication’s adverse effects; fear of partners mistrust regarding HIV status; and anxiety about being perceived as HIV-positive. Regarding testing history, 86.50% of participants had undergone HIV testing, while 6.33% reported previous STD infections (Table 2).

**Table 2:** Sexual health care of MSM in Maanshan City, China.

Variable	Subgroup	Frequency	Percentage (%)
HIV awareness (n = 837)	No	60	7.17
	Yes	777	92.83
PrEP awareness (n = 837)	No	647	77.30
	Yes	190	22.70
PrEP willingness (n = 837)	No/Probably	88	10.51
	Yes	749	89.49
The way of taking PrEP (n = 749)			
Willingness to take PrEP every day	No	611	81.58
	Yes	138	18.42
Willingness to take PrEP on-demand	No	41	5.47
	Yes	708	94.53
Payment methods for PrEP (n = 749)			
For free	No	48	6.41
	Yes	701	93.59
For a fee	No	241	32.18
	Yes	508	67.82
By health insurance	No	256	34.18
	Yes	493	65.82
Reasons for unwilling to take PrEP (n = 88)			
Worry about adverse effects of PrEP	No	11	12.50
	Yes	77	87.50
Sexual partners would get angry at my mistrust if I take the PrEP	No	48	54.55
	Yes	40	45.45
People would look at me as an HIV infector if I take PrEP	No	67	76.14
	Yes	21	23.86
Ever tested for HIV (n = 837)	No	113	13.50
	Yes	724	86.50
Infected STD in the last 12 months (n = 837)	No	784	93.67
	Yes	53	6.33

### Factors that influence awareness and willingness to use PrEP

Univariate analysis revealed several characteristics significantly associated with a willingness to use PrEP, including education level, marital status, sexual role, history of casual sex and UAI in the previous six months, current regular male sexual partner status, and PrEP awareness. The multivariate logistic regression model showed that MSM with vocational school education were significantly more likely to use PrEP (aOR = 1.91, 95% CI:1.00,3.63). Similarly, those reporting casual sex in the previous six months demonstrated higher PrEP usage likelihood (aOR = 2.13, 95% CI:1.27,3.56). PrEP awareness was strongly associated with increased usage (aOR = 4.96, 95% CI:1.75,14.04), while recent UAI was associated with decreased usage (aOR = 0.29, 95% CI:0.17,0.48) (Table 3).

**Table 3:** Logistic regression analysis of factors associated with willingness to use PrEP.

Characteristics	Level	N(%)	Univariate analysis		Multivariate analysis	
			cOR^a^(95%CI)	p-value	aOR^b^(95%CI)	p-value
Age, years	18-25	255(0.90)	Reference			
	26-30	209(0.88)	0.85(0.49,1.47)	0.560		
	＞30	285(0.90)	1.05(0.61,1.78)	0.870		
Education	High school or below	341(0.87)	Reference		Reference	
	Vocational school	235(0.94)	2.39(1.31,4.34)	0.004	1.91(1.00,3.63)	0.049
	University or above	173(0.89)	1.26(0.73,2.15)	0.407	1.11(0.61,2.00)	0.741
Marital status	Never married	470(0.91)	Reference		Reference	
	Married	225(0.86)	0.60(0.38,0.94)	0.027	0.85(0.50,1.45)	0.549
	Divorced/Widowed	54(0.92)	1.06(0.40,2.77)	0.910	1.30(0.45,3.77)	0.626
Monthly personal income, RMB	<1000	103(0.91)	Reference			
	1000-2999	267(0.90)	0.84(0.40,1.77)	0.639		
	≥3000	379(0.89)	0.78(0.38,1.60)	0.503		
Sexual orientation	Gay	733(0.90)	Reference		Reference	
	Heterosexual/Bisexual/Unsure	16(0.76)	0.36(0.13,1.01)	0.053	0.41(0.13,1.25)	0.118
Sexual role	Top/Insertive	231(0.87)	Reference		Reference	
	Bottom/Receptive	124(0.84)	0.78(0.45,1.38)	0.394	0.94(0.51,1.75)	0.854
	Versatile	394(0.93)	2.06(1.23,3.46)	0.006	1.66(0.95,2.87)	0.073
Whether currently have a regular male sexual partner	No	387(0.93)	Reference		Reference	
	Yes	362(0.86)	0.48(0.30,0.77)	0.002	0.68(0.40,1.15)	0.152
Age of first anal sex with men		837(1.00)	0.98(0.93,1.02)	0.306		
Number of anal sex acts in the previous month		837(1.00)	0.96(0.87,1.06)	0.449		
Casual sex in the last 6 months	No	191(0.81)	Reference		Reference	
	Yes	558(0.93)	3.06(1.95,4.79)	<0.001	2.13(1.27,3.56)	0.004
UAI in the last 6 months	No	502(0.95)	Reference		Reference	
	Yes	247(0.81)	0.24(0.15,0.39)	<0.001	0.29(0.17,0.48)	<0.001
HIV awareness	No	55(0.92)	Reference			
	Yes	694(0.89)	0.76(0.30,1.95)	0.569		
Ever tested for HIV	No	97(0.86)	Reference			
	Yes	652(0.90)	1.49(0.83,2.67)	0.117		
STD diagnosis last 12 months	No	698(0.89)	Reference			
	Yes	51(0.96)	3.14(0.75,13.13)	0.117		
PrEP awareness	No	563(0.87)			Reference	
	Yes	186(0.98)	6.94(2.51,19.18)	<0.001	4.96(1.75,14.04)	0.003

^a^: cOR: crude Odds Ratio.

^b^: aOR: adjusted Odds Ratio.

Regarding PrEP awareness, univariate analysis identified significant associations with age, education, monthly income, sexual orientation, sexual role, current regular male sexual partner, recent casual sex history, and HIV testing history. Multivariate analysis demonstrated that university-educated MSM had significantly higher PrEP awareness (aOR = 2.27, 95% CI:1.46,3.53). Compareed to Top/Insertive partners, versatile individuals showed greater PrEP knowledge (aOR = 1.57, 95% CI:1.04,2.37). Those with recent casual sex experience (aOR = 1.64, 95% CI:1.04,2.57) and previous HIV testing (aOR = 2.38, 95% CI:1.22,4.66) were also more likely to be PrEP-aware (Table 4).

**Table 4:** Logistic regression analysis of factors associated with awareness of PrEP.

Characteristics	Level	N(%)	Univariate analysis		Multivariate analysis	
			cOR^a^(95%CI)	p-value	aOR^b^(95%CI)	p-value
Age, years	18-25	80(0.28)	Reference		Reference	
	26-30	48(0.20)	0.65(0.43,0.97)	0.037	0.70(0.43,1.13)	0.143
	＞30	62(0.20)	0.62(0.43,0.91)	0.014	0.72(0.44,1.17)	0.185
Education	High school or below	67(0.17)	Reference		Reference	
	Vocational school	55(0.22)	1.37(0.92,2.04)	0.119	1.17(0.76,1.78)	0.481
	University or above	68(0.35)	2.63(1.77,3.90)	<0.001	2.27(1.46,3.53)	<0.001
Marital status	Never married	126(0.24)	Reference	0.313		
	Married	53(0.20)	0.78(0.55,1.13)	0.190		
	Divorced/Widowed	11(0.19)	0.71(0.36,1.41)	0.326		
Monthly personal income, RMB	<1000	38(0.34)	Reference		Reference	
	1000-2999	52(0.17)	0.42(0.26,0.68)	<0.001	0.65(0.36,1.15)	0.139
	≥3000	100(0.23)	0.61(0.39,0.95)	0.029	0.93(0.52,1.67)	0.818
Sexual orientation	Gay	189(0.23)	Reference		Reference	
	Heterosexual/Bisexual/Unsure	1(0.05)	0.17(0.02,1.24)	0.081	0.17(0.02,1.31)	0.090
Sexual role	Top/Insertive	45(0.17)	Reference	0.001	Reference	
	Bottom/Receptive	26(0.18)	1.05(0.62,1.78)	0.866	0.99(0.57,1.74)	0.986
	Versatile	119(0.28)	1.92(1.31,2.82)	0.001	1.57(1.04,2.37)	0.031
Whether currently have a regular male sexual partner	No	108(0.26)	0.69(0.50,0.96)	0.028	Reference	
	Yes	82(0.20)	Reference		0.81(0.56,1.17)	0.271
Age of first anal sex with men		837(1.00)	0.99(0.96,1.03)	0.784		
Number of anal sex acts in the previous month		837(1.00)	0.98(0.90,1.07)	0.627		
Casual sex in the last 6 months	No	35(0.15)	Reference		Reference	
	Yes	155(0.26)	2.00(1.33,2.99)	0.001	1.64(1.04,2.57)	0.031
UAI in the last 6 months	No	131(0.25)	Reference		Reference	
	Yes	59(0.19)	0.73(0.52,1.03)	0.074	0.79(0.54,1.16)	0.236
HIV awareness	No	16(0.27)	Reference			
	Yes	174(0.22)	0.79(0.44,1.44)	0.447		
Ever tested for HIV	No	11(0.10)	Reference		Reference	
	Yes	179(0.25)	3.05(1.60,5.80)	0.001	2.38(1.22,4.66)	0.011
STD diagnosis last 12 months	No	173(0.22)	Reference		Reference	
	Yes	17(0.32)	1.67(0.91,3.04)	0.095	1.48(0.78,2.80)	0.227

^a^: cOR: crude Odds Ratio.

^b^: aOR: adjusted Odds Ratio.

## Discussion

MSM of Maanshan City showed higher PrEP willingness (89.49%) but lower awareness (22.7%) compared to 2021 global averages (58.6% willingness and 50.0% awareness) [25]. While PrEP use among Chinese high-risk groups increased since WHO ‘s 2012 recommendation, awareness remains low [26,27]. The local usage rate (5.4%) was about 1/6 of Washington State MSM rates during the same period [28]. China’s delayed PrEP approval and limited pilot implementation, combined with variable awareness and complex influencing factors among high-risk populations, contribute to this disparity.

Similarly, one study found 66% of participants knew about PrEP, but only 10% had used it [29], highlighting a concerning awareness-uptake gap. Bridging this gap is crucial for effective HIV prevention [30]. Future PrEP promotion should emphasize both continued condom use and accurate information about PrEP’s safety and effectiveness.

Side effects were a major concern (87.5%). Evidence shows TDF may cause reversible bone mineral density decreases in young African women [31], though other factors like contraception and pregnancy may contribute [32,33]. Tenofovir fumarate and emtricitabine (TDF-FTC) users reported bone density changes and gastrointestinal symptoms, though long-term effects remain uncertain [34]. Importantly, meta-analyses show minimal resistance risk and clear HIV protection benefits [35]. While the long-term toxic potential of TDF-FTC requires further, we must ultimately weigh safety concerns against the proven benefits of HIV-1 prevention [5]. As we await an effective HIV vaccine, TDF-FTC PrEP in high-risk populations may help reduce HIV infection [36]. The public health risks are outweighed by the prevention advantages of TDF-based PrEP [37], with benefits extending beyond individuals to slowing HIV spread at population level [38].

The finding that 45.45% feared partner anger and 23.86% experienced medication discrimination suggests PrEP users may be mislabeled as HIV-positive due to taking HIV-related drugs. These misunderstandings hinder PrEP promotion and harm MSM health. Some view PrEP as shameful, associating it with “less honorable” casual sex practices. When risk assessment tools categorize multiple partners and behaviors as high risk, they inadvertently reinforce PrEP stigma [39]. However, over 90% reported their MSM peers were open to PrEP use [40]. Without stigma reduction through improved HIV prevention awareness [41], these attitudes may block PrEP acceptance among high-risk individuals. Community leaders can combat stigma by discussing PrEP openly [42], leveraging digital platforms to educate MSM about PrEP while reducing associated stigma. Social marketing, community engagement, and policy analysis may enhance PrEP acceptability, reduce stigma, and expand treatment access [43]. Future publicity should improve PrEP knowledge while reshaping perceptions of HIV and prevention.

Among the acceptable delivery methods of PrEP, 701 (93.59%) MSM preferred free distribution, 508 (67.82%) were willing to pay for PrEP and 493 (65.82%) wanted it to be covered by health insurance. These findings align with a study conducted among MSM in Chengdu [44]. According to research from South Korea, the primary barrier to PrEP uptake (32%) was lack of insurance coverage. In the United States, PrEP cost can be subsidized through various programs [45], but data indicate low PrEP usage among uninsured men [46]. TDF and TDF-FTC are already widely accessible, and on-demand PrEP has been approved in France on IPERGAY trial data [47]. By significantly reducing HIV incidence among MSM, on-demand PrEP expands patients options [48]. However, in China, 33.8% of respondents still cited “health insurance” as a concern, highlighting its influence on PrEP accessibility [7]. Currently, PrEP is not free in China, with monthly cost reaching approximately $300 — a substantial financial burden for individuals [49]. Modeling suggests PrEP could avoid economic costs of 17,277–18,452$/quality-adjusted life year (QALY) in China [50]. PrEP pricing should consider the target population’s economic capacity, with efforts to negotiate lower drug prices, include PrEP in medical insurance coverage, or provide subsidies to those in need.

Logistic regression analysis revealed that versatile sexual behavior, casual sex in the last 6 months, university-level education or higher, and prior HIV testing served as protective factors for PrEP awareness. Notably, versatile and casual sex in the last 6 months were also significant risk factors for HIV infection among the MSM, suggesting these individuals may possess heightened PrEP awareness as a prevention measure [42]. Although overall PrEP awareness was lower in Maanshan, higher-educated campaigns for MSM were more likely to recognize its importance. Future efforts could prioritize awareness and education campaigns for MSM with lower educational attainment. Additionally, those who had previously tested for HIV demonstrated better PrEP understanding, indicating that health care services like HIV testing present key opportunities to disseminate PrEP knowledge.

The research findings indicated that MSM individuals who had engaged in UAI within the past 6 months showed lower willingness to accept PrEP, suggesting reduced self-protection awareness in this group. On the contrary, MSM with stronger PrEP awareness demonstrated greater willingness to use it. As one study confirmed, awareness positively correlates with willingness [9]. This implies that targeted education campaigns for key populations with low protection awareness could enhance PrEP acceptance among MSM.

The Joint United Nations Programme on HIV/AIDS (UNAIDS) and World Health Organization (WHO) endorse the undetectable equals nontransmittable (U = U) concept and recommend its dissemination among vulnerable groups [51]. While U = U is widely recognized in Latin America’s HIV community, it remains poorly understood and trusted in China [52]. U = U is the maintenance of viral load <200 copies/mL plus adherence to ART, allowing viral load control and zero transmission [53]. This concept has been shown to boost both PrEP awareness and acceptance among MSM, while alleviating HIV-related fear and stigma. Therefore, Chinese healthcare institutions should integrate U = U messaging into HIV education programs before intensifying prevention awareness campaigns for MSM.

This study had several limitations. Firstly, the relatively small sample size may limit generalizability to China’s broader MSM population. Second, the RDS methodology potentially introduced bias, as participants recruited through MSM-friendly organizations tended to be younger and more educated. Consequently, actual PrEP awareness and willingness levels may be lower than reported. Third, while focusing on MSM, the study did not address other high-risk populations like drug users and sex workers. Future research should explore PrEP awareness among all HIV-vulnerable populations to better inform prevention strategies.

## Conclusion

MSM who are unwilling to use PrEP primarily cite self-payment costs and potential side effects as their main reasons for refusal. Reducing the financial burden of PrEP for MSM, promoting on-demand PrEP usage, and expanding HIV and PrEP education could help increase PrEP acceptance. Strengthening sexual health promotion and improving access to HIV testing may further enhance PrEP awareness among MSM. Additionally, combining condom use with PrEP can more effectively prevent HIV transmission in this population.

## Supporting information

S1 File De-identified survey responses from study participants. (XLSX)
